# Reduction of miR-29c enhances pancreatic cancer cell migration and stem cell-like phenotype

**DOI:** 10.18632/oncotarget.3089

**Published:** 2014-12-30

**Authors:** Jianxin Jiang, Chao Yu, Meiyuan Chen, Hao Zhang, Se Tian, Chengyi Sun

**Affiliations:** ^1^ Department of Hepatobiliary Surgery, Affiliated Hospital of Guiyang Medical College, Guiyang, Guizhou, China

**Keywords:** Pancreatic cancer, miR-29c, Wnt, FRAT2, LRP6

## Abstract

The hallmarks of pancreatic cancer are limitless replicative potential as well as tissue invasion and metastasis, leading to an extremely aggressive disease with shockingly *high* lethality. However, the molecular mechanisms underlying these characteristics remain largely unclear. Herein, we report the results of a differential miRNA expression screen that compared pancreatic cancer tissues and normal pancreatic tissues, where the pancreatic cancer tissues had highly downregulated miR-29c with relative Wnt cascade hyperactivation. MiR-29c directly suppressed the following Wnt upstream regulators: frequently rearranged in advanced T-cell lymphomas 2 (FRAT2), low-density lipoprotein receptor–related protein 6 (LRP6), Frizzled-4 (FZD4) and Frizzled-5 (FZD5). Furthermore, transforming growth factor-β (TGF-β) inhibited miR-29c expression, leading to Wnt activation. Significantly, our results were consistent with an important correlation between miR-29c levels and TGF-β hyperactivation and the activated Wnt cascade in human pancreatic cancer specimens. These findings reveal a novel mechanism for Wnt hyperactivation in pancreatic cancer and may suggest a new target for clinical intervention in pancreatic cancer.

## INTRODUCTION

Pancreatic cancer (PANC), which caused 330,000 deaths globally in 2012 [1], has the worst 1- and 5-year survival rates of all cancers. The 1-year relative survival rate for all stages of PANC combined is approximately 25%, and 5-year survival is estimated as no more than 6% [2, 3]. Patients with PANC have poor prognosis and short survival partly because PANC usually causes no symptoms in the early stage, leading to locally advanced or metastatic disease at the time of diagnosis [4, 5]. Unfortunately, the lack of effective therapy worsens the situation [6]. Therefore, there is an urgent need to design novel strategies for achieving better treatment outcome in patients diagnosed with PANC.

Metastasis and recurrence are the two main causes of cancer-related mortality. An increasing number of observations suggest that the stemness of cancer cells is implicated in tumor progression and metastasis [7-10]. Although a series of studies has proven the cancer stem cell hypothesis in PANC [11-15], the precise molecular mechanisms underlying the stem cell-like properties of PANC cells remain largely unknown. Undercover molecular mechanisms that collaboratively regulate PANC cell metastasis and stemness are expected to provide new insights into the development of novel and effective therapy for PANC. In this context, identifying genetic and/or epigenetic factors that modulate the stem cell-like phenotype of PANC cells is of significant importance in the clinic.

MicroRNA (miRNA) dysregulation is implicated in the development and progression of practically all tumor types [16]. MiRNAs may influence cancer cell stemness particularly by modulating specific pathways [17-20]. The canonical Wnt cascade modulates both self-renewal and oncogenesis in different organs [8, 21] and is hyperactive in PANC [22]. Herein, we reveal that following inhibition by transforming growth factor-β (TGF-β)/Smad3 signaling, miR-29c was remarkably decreased in PANC cells. Reintroducing miR-29c into PANC cells significantly suppressed the PANC cell malignant phenotype and the stem cell–like phenotype by targeting Wnt signaling pathway regulators. Furthermore, the *xenograft* tumor *model* showed that miR-29c inhibited PANC tumorigenesis *in vivo*. Our findings reveal a novel pathway by which epigenetic modulation of miR-29c renders the PANC cell abilities of robust self-renewal and aggressive metastasis ineffective.

## RESULTS

### Reduced miR-29c levels in PANC correlated with patient prognoses

**Figure 1:**
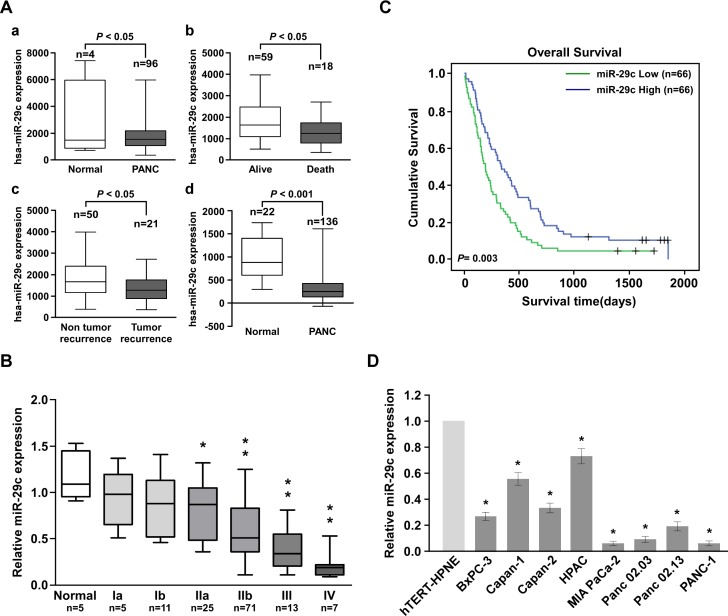
Reduced miR-29c expression in pancreatic cancer with poor prognosis A, Expression profiles of miRNA were obtained from the TCGA database and the NCBI GEO database. B, Statistical analysis of miR-29c expression in normal pancreas tissues (n = 5) and pancreatic cancer specimens of different WHO grades (n = 132). C, Kaplan-Meier curves of pancreatic cancer patients with low- versus high-expression of miR-29c (n = 132; P = 0.003). D, Real-time PCR analysis of miR-29c expression in 8 indicated pancreatic cancer cell lines and normal pancreas cell line hTERT-HPNE.

We retrieved miRNA expression profiles from two data sets: TCGA and the Gene Expression Omnibus dataset GSE24279 [23], and found that miR-29c was significantly decreased in PANC tissues compared with normal pancreatic tissues. Interestingly, miR-29c was also downregulated in patients who had died from PANC, as well as patients with proven tumor recurrence (Figure 1A). To validate the expression pattern of miR-29c in PANC, quantitative reverse transcription-PCR was conducted on five normal pancreatic tissue samples and 132 frozen PANC samples. MiR-29c levels remained high in grade I tumors but became markedly lower in grade IIb tumors and were further decreased in grade III and IV tumors (Figure 1B). Statistical analysis revealed that miR-29c was associated with shorter overall survival (*P* = 0.003) (Figure 1C). Additionally, miR-29c expression was reduced in the eight PANC cell lines tested compared with that in the normal hTERT-HPNE cell line (Figure 1D). These findings suggest a possible link between miR-29c reduction and human PANC progression.

### Restoring miR-29c suppressed PANC cell migration and invasion and attenuated the stem cell–like phenotype

**Figure 2:**
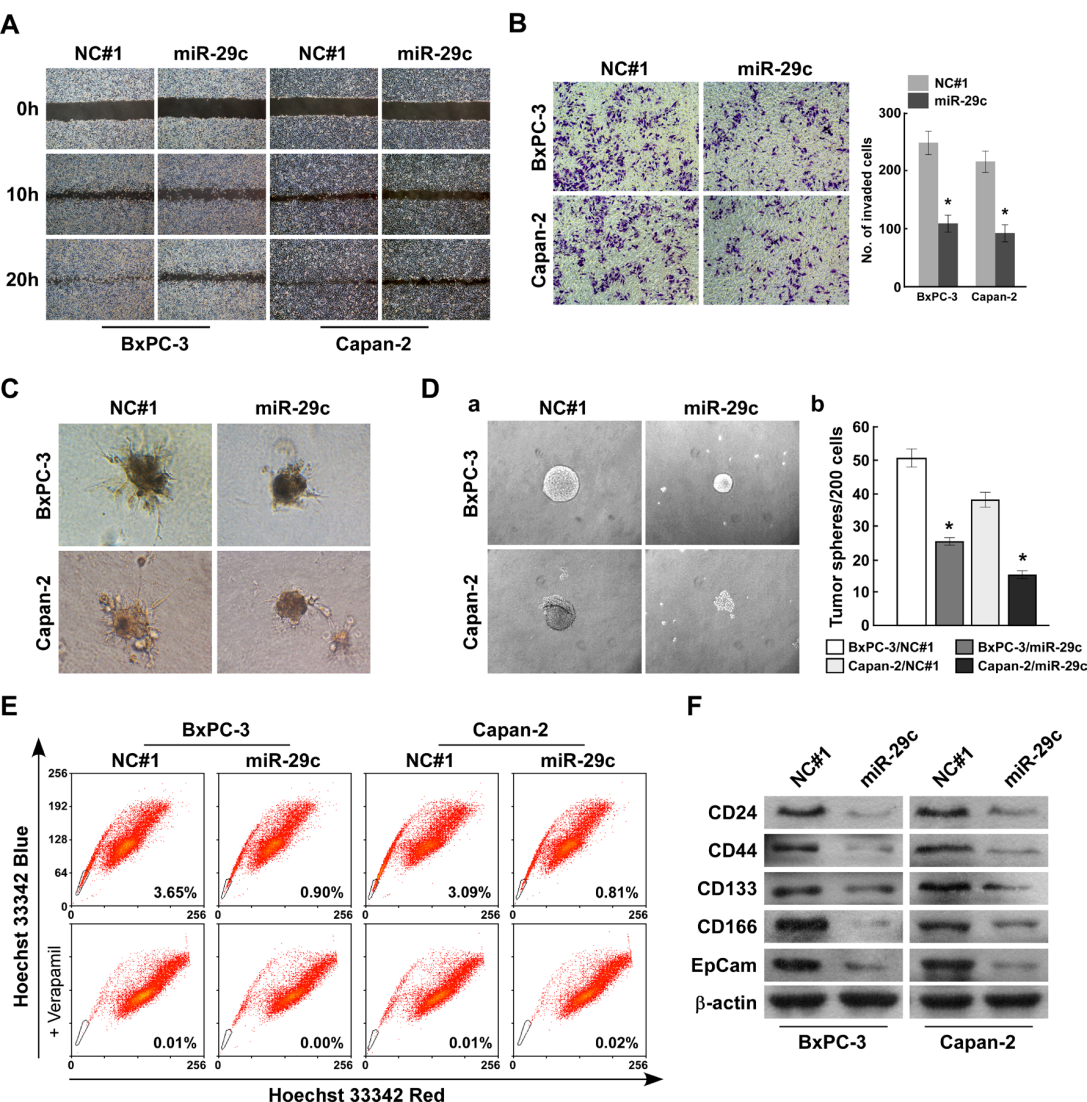
MiR-29c suppresses pancreatic cancer cells migration and invasion as well as attenuates stem cell-like phenotype *in vitro* A, Wound-healing assay was conducted with indicated cells and images were taken at 0, 10, and 20 hours. B, Representative images (left) and quantification (right) of penetrated cells were analyzed using the Transwell matrix penetration assay. C, Representative micrographs of indicated cultured cells at day 7 of culture in 3-dimensional spheroid invasion assay. D, Representative images of spheres formed by the indicated cells (left) and histograms showing the mean number of spheres (right). E, Hoechst 33342 dye exclusion assay showing that overexpressing miR-29c attenuated the SP cells in the indicated cells. F, Western blot analysis of CD24, CD44, CD133, CD166 and EpCam expression in the indicated cells. β-actin was used as a loading control. Error bars represent mean ± SD from 3 independent experiments; *, *P* < 0.05; **, *P* < 0.01.

We selected the BxPC-3 and Capan-2 PANC cell lines to investigate whether miR-29c could modulate PANC cell migration and invasiveness. A wound-healing assay was used to detect the effect of miR-29c on cell migration. Compared with the negative control #1 (NC#1) cells, which spread to the center within 20 hours, miR-29c-transfected cells exhibited clearly slower migration and decreased cell spreading (Figure 2A). We used the Transwell invasion assay to determine the effect of miR-29c expression on PANC cell invasion. Compared with the control cells, fewer miR-29c–transfected cells invaded across the Matrigel-precoated membrane (Figure 2B). Significantly, the 3-dimensional spheroid invasion assay revealed that NC#1-control cells displayed highly aggressive invasive growth after 7 days, but the miR-29c-transfected-cells did not (Figure 2C). Taken together, these findings indicate that miR-29c greatly suppresses PANC cell migration and invasion.

Cancer tends to relapse after surgical resection, and this characteristic is considered mainly ascribable to the stem cell-like properties of a fraction of cells within the tumor [24]. As miR-29c levels are particularly decreased in patients with proven tumor recurrence, we suggest the potential role of miR-29c in the development and maintenance of the stem cell-like property of PANC cells. Compared with control cells, miR-29c-transfected cells formed much smaller spheres after 7 days of culture (Figure 2D). The Hoechst 33342 dye exclusion assay showed that miR-29c overexpression decreased the proportion of side population-positive (SP+) cells from 3.65% to 0.90% in BxPC-3 cells, and from 3.09% to 0.81% in Capan-2 cells (Figure 2E). Furthermore, the CD44+ population and the expression of multiple pluripotency-associated factors were dramatically decreased in miR-29c-transfected cells compared with the control cells (Figure 2F). Collectively, our results reveal that miR-29c restoration attenuates the PANC cell self-renewal ability.

### MiR-29c upregulation attenuated tumorigenicity and invasion *in vivo*

**Figure 3:**
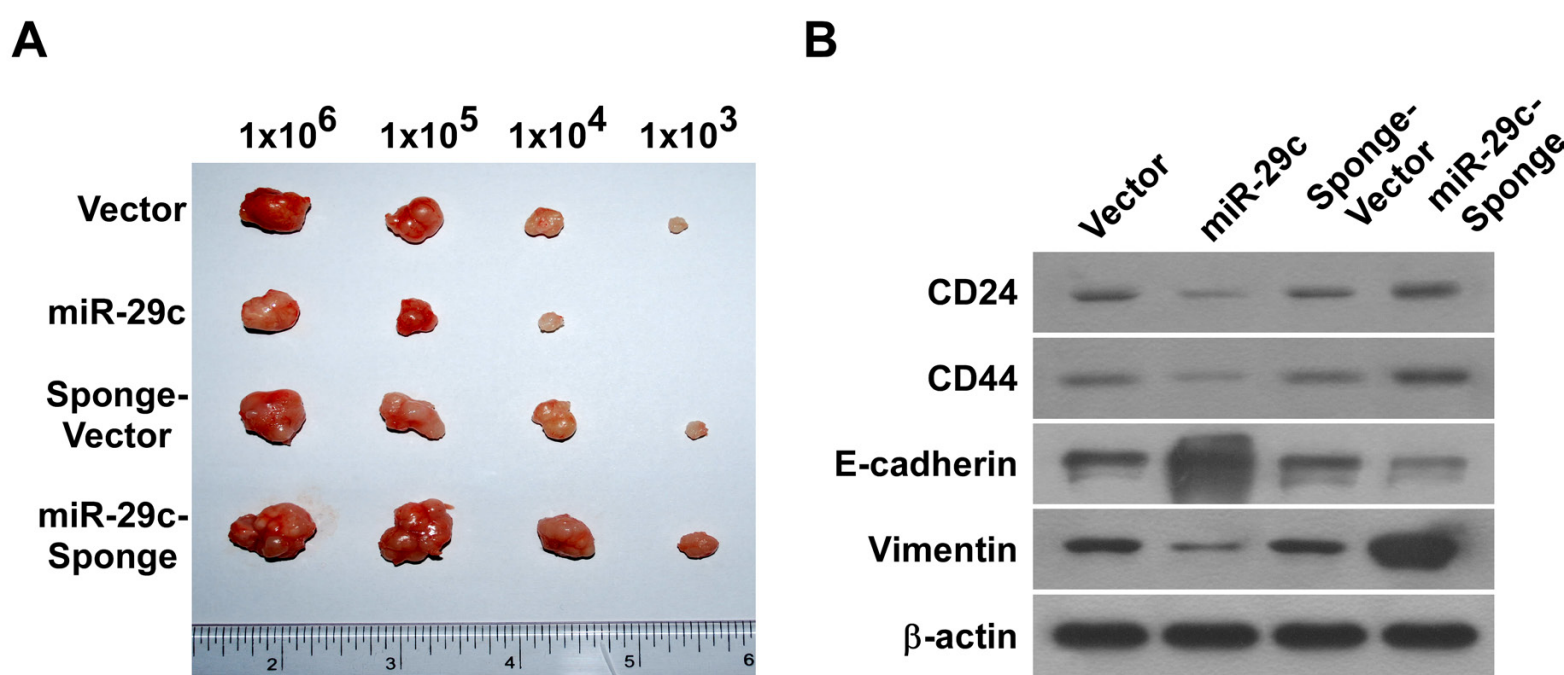
MiR-29c upregulation attenuated tumorigenicity and invasion *in vivo* A, The tumors formed by miR-29c-transduced BxPC-3 cells were smaller than the vector control tumors. Conversely, the tumors formed by miR-29c-sponge-transduced BxPC-3 cells were larger than the tumors formed by the sponge-vector cells. Representative images of the tumors are shown. B, Western blot analysis of CD24, CD44, E-cadherin and vimentin expression in the indicated tissue samples. β-actin was used as a loading control.

To understand whether miR-29c is involved in PANC cell tumorigenesis and invasiveness *in vivo*, we subcutaneously inoculated 10^3^–10^6^ cells into the inguinal folds of nude mice. The tumors formed by miR-29c-transduced PANC cells were visibly smaller than the vector control tumors (Figure 3A). We used a stable miRNA sponge strategy to inhibit miR-29c *in vivo*, and the inguinal tumors formed by miR-29c-sponge-transduced cells were clearly larger than the sponge-vector control tumors. Moreover, immunoblotting showed that reintroducing miR-29c decreased the expression levels of multiple pluripotency-associated factors (CD24, CD44) and mesenchymal factor (vimentin) *in vivo*, while inhibiting miR-29c increased them, but the opposite was true for epithelial factor (E-cadherin) expression levels (Figure 3B). Our data validate the premise that reintroducing miR-29c suppresses the tumorigenic and invasive behavior of PANC *in vivo*.

### MiR-29c suppressed Wnt signaling

**Figure 4:**
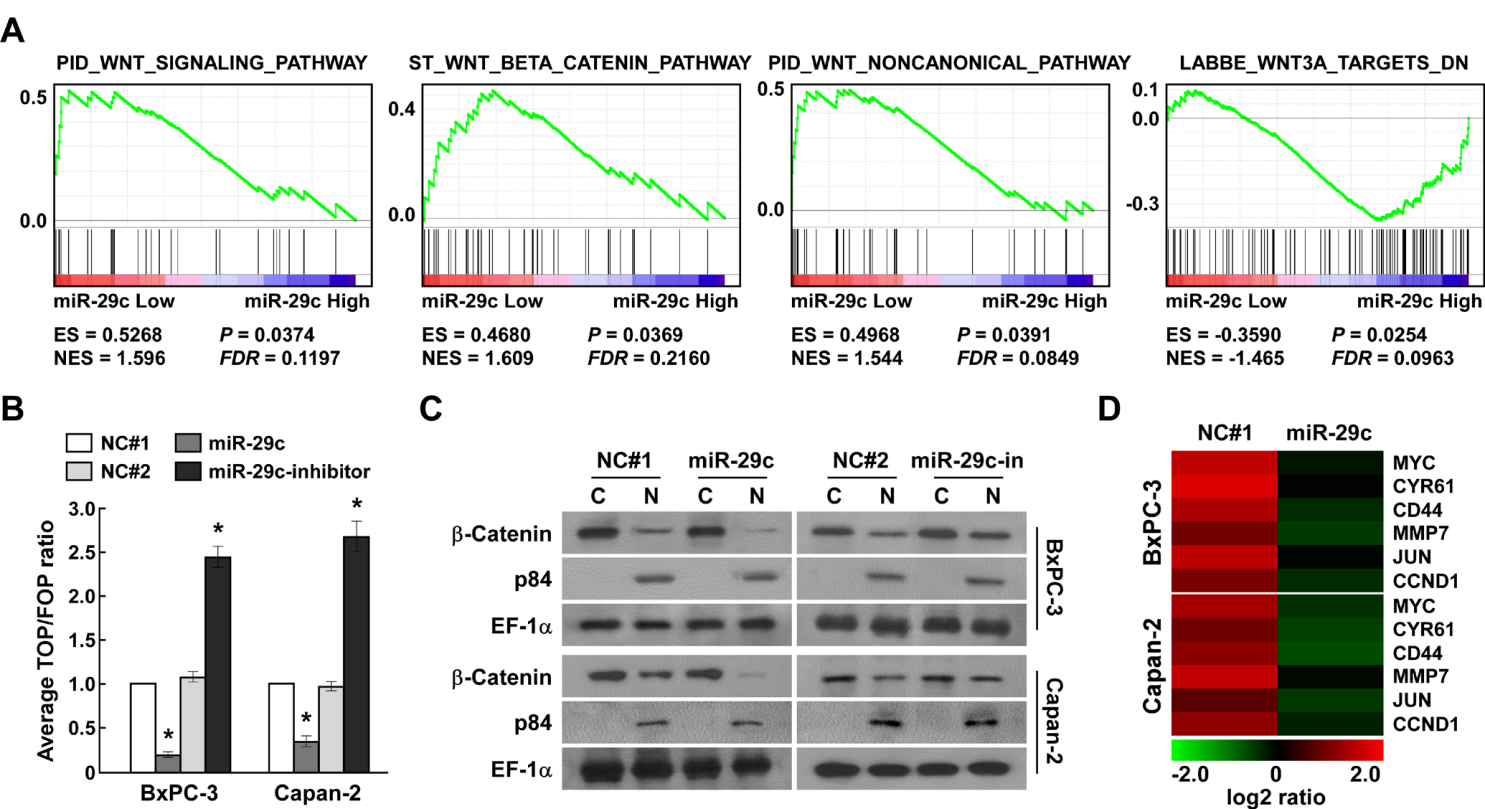
MiR-29c suppresses Wnt signaling A, GSEA plot showing that miR-29c expression is inversely correlated with Wnt-activated gene signatures and positively correlated with Wnt-suppressed gene signatures in published pancreatic cancer patient gene expression profiles (TCGA, *n* = 96). B, TOP/FOP luciferase ratio reported Wnt/β-catenin pathway activity in the indicated cells. C, Western blotting analysis of β-catenin expression in the cytoplasm (C) and nucleus (N) of the indicated cells. Nuclear protein p84 was used as a nuclear protein marker and EF-1α as a loading control. D, Relative mRNA expression of Wnt/β-catenin-regulated genes in the indicated cells was assessed by real-time PCR. *GAPDH* was used as a loading control.

The Wnt cascade has been recognized to play a critical role in regulating self-renewal and oncogenesis as well as metastasis [25, 26]. We analyzed the expression of miR-29c and Wnt-regulated gene signatures via GSEA [27] in published PANC patient expression profiles, finding that miR-29c expression levels were inversely correlated with the Wnt-activated gene signatures and positively correlated with Wnt-suppressed gene signatures (Figure 4A). We next tested whether miR-29c suppressed Wnt signaling. β-Catenin/T cell factor (TCF) activity was significantly decreased in miR-29c-overexpressing PANC cells, but was increased in miR-29c–inhibited cells (Figure 4B). Furthermore, cellular fractionation showed that miR-29c overexpression decreased nuclear accumulation of β-catenin (Figure 4C), indicating that miR-29c suppresses the Wnt/β-catenin pathway by decreasing nuclear β-catenin accumulation. Real-time PCR analyses of miR-29c-overexpressing PANC cells revealed that the expression levels of six classically recognized Wnt/β-catenin target genes were remarkably decreased (Figure 4D). In summary, our findings indicate that miR-29c augments PANC tumorigenicity and invasion by suppressing Wnt signaling.

### MiR-29c directly suppressed Wnt cascade–activated regulatory genes

**Figure 5:**
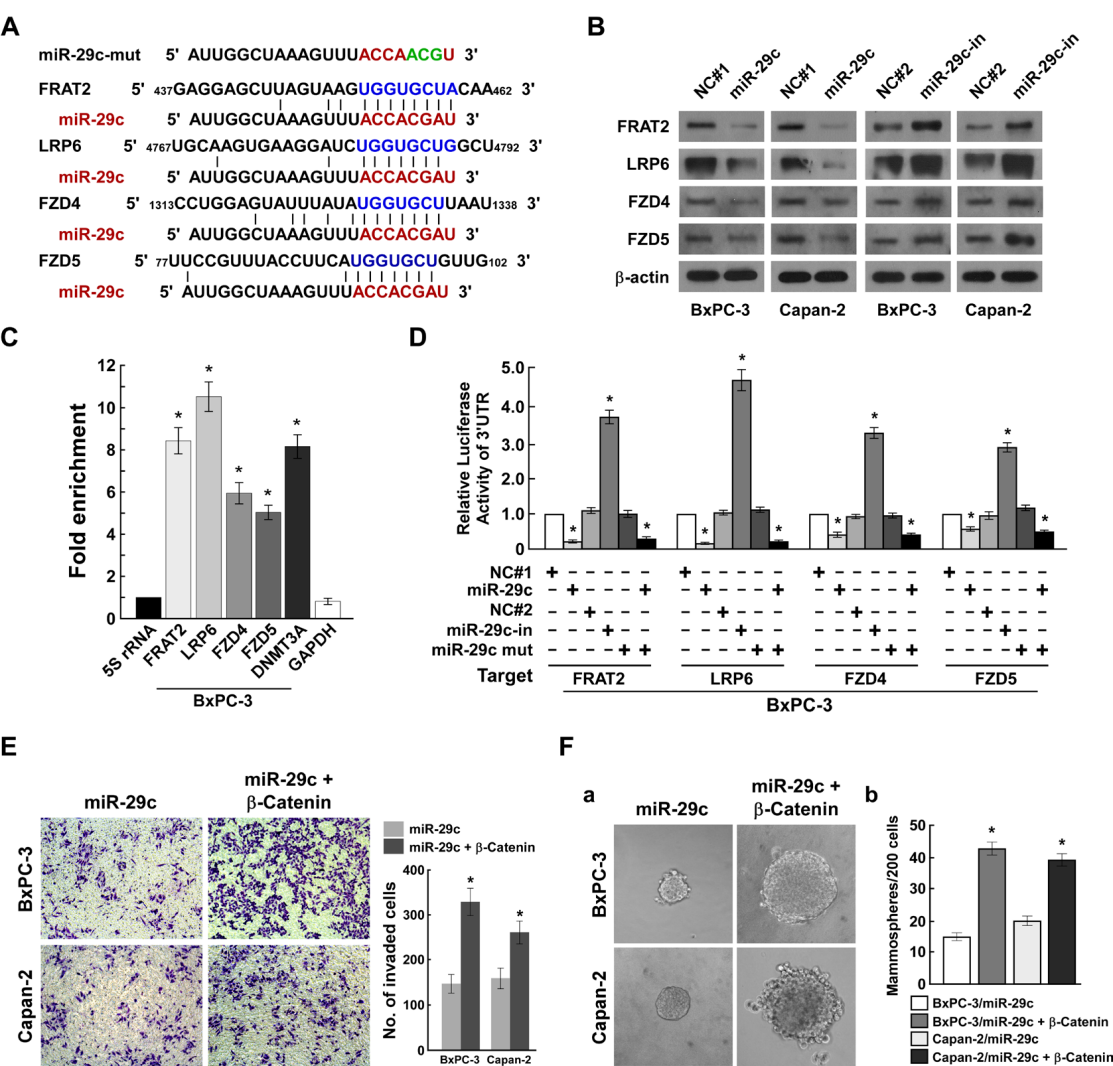
MiR-29c directly suppresses multiple Wnt cascade activate regulatory genes A, Predicted miR-29c target sequences in FRAT2-3′UTR, LRP6-3′UTR, FZD4-3′UTR and FZD5-3′UTR. B, Western blot analysis of FRAT2, LRP6, FZD4 and FZD5 expression in the indicated cells. β-actin served as the loading control. C, miRNP IP assay showed association of miR-29c with *FRAT2*, *LRP6, FZD4, FZD5, DNMT3A* and *GAPDH* were used as positive and negative controls, respectively, and *5S* rRNA was used as a control for overall expression levels. D, Luciferase activities of FRAT2-3′UTR, LRP6-3′UTR, FZD4-3′UTR or FZD5-3′UTR in vector- or miR-29c-transduced cells, or in miR-29c-transduced cells transfected with miR-29c-mut, or in vector-transduced cells transfected with NC or miR-29c inhibitor. E, Representative images (left) and quantification (right) of penetrated cells were analyzed using the Transwell matrix penetration assay. F, Representative images of spheres formed by the indicated cells (left) and histograms showing the mean number of spheres (right). Error bars represent mean ± SD from 3 independent experiments; *, *P* < 0.05; **, *P* < 0.01.

As miRNAs exert their functions by targeting multiple transcripts, we screened for targets of miR-29c using TargetScan, identifying four potential targets (*FRAT2*, *LRP6*, *FZD4*, *FZD5*) (Figure 5A). Immunoblotting analysis showed that miR-29c overexpression repressed *FRAT2*, *LRP6*, *FZD4* and *FZD5* expression levels, while miR-29c inhibition increased them (Figure 5B). The microribonucleoprotein immunoprecipitation and luciferase activity assays demonstrated that miR-29c associated directly with the 3′ UTR of *FRAT2*, *LRP6*, *FZD4* and *FZD5* (Figure 5C, 5D and Supplementary Figure S1A,1B). As *FRAT2*, *LRP6*, *FZD4* and *FZD5* are the upstream regulatory genes of Wnt signaling, we assumed that exogenous β-catenin expression would restore the invasive and carcinogenic ability of miR-29c-overexpressing PANC cells, which our findings validated (Figure 5E, 5F). Taken together, our data show that miR-29c inhibits PANC tumorigenicity and invasion through direct suppression of multiple Wnt signaling core regulatory genes.

### TGF-β/Smad3 signaling inhibited miR-29c in PANC

**Figure 6:**
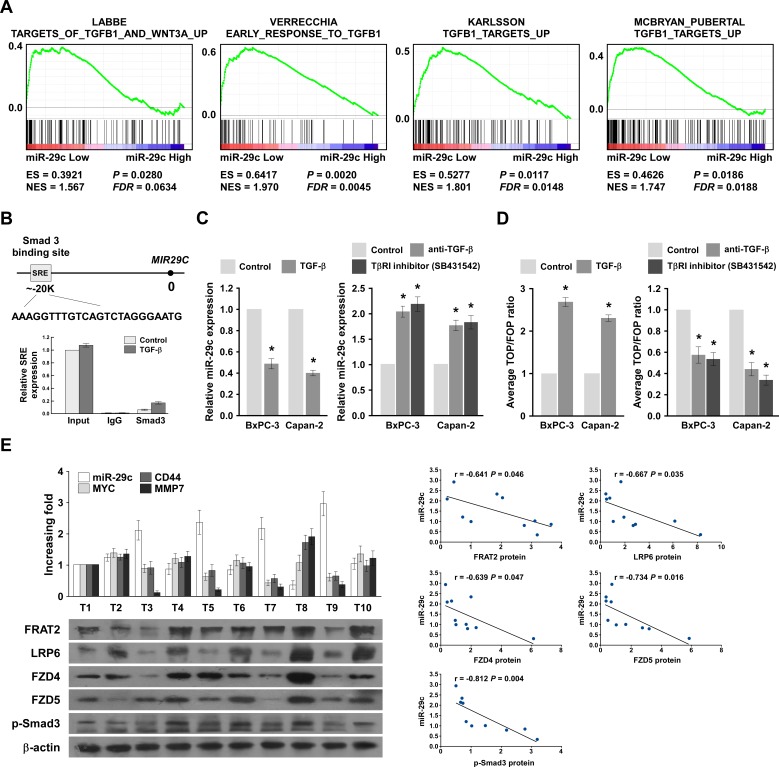
TGF-β/Smad3 inhibits miR-29c expression and clinical relevance of the TGF-β/Smad3/miR-29c/Wnt axis in pancreatic cancer A, GSEA plot showing that miR-29c expression is inversely correlated with TGF-β-activated gene signatures in published pancreatic cancer patient gene expression profiles (TCGA, *n* = 96). B, Schematic of typical SRE of the *MIR29c* promoter. Also shown are ChIP assay results for the SRE of *MIR29c* promoter physically associated with Smad3. C, Real-time PCR analysis of miR-29c expression in the indicated cells, either treated with 100 pM TGF-β for 3 hours or treated with or without the TβRI inhibitor (2 μM) or a neutralizing anti-TGF-β antibody (2 μg/ml) for 3 hours. Transcript levels were normalized by *U6* expression. D, TOP/FOP luciferase ratio reported Wnt/β-catenin pathway activity in the indicated cells, either treated with 100 pM TGF-β for 3 hours or treated with or without the TβRI inhibitor (2 μM) or a neutralizing anti-TGF-β antibody (2 μg/ml) for 3 hours. E, Expression and correlation of miR-29c with *CD44*, *MYC*, and *MMP7* mRNA expression, as well as FRAT2, LRP6, FZD4, FZD5 and p-Smad3 protein expression in 10 freshly collected pancreatic cancer samples.

We explored the molecular mechanism that mediates the reduction of miR-29c in PANC cells, using Genomic Identification of Significant Targets in Cancer (GISTIC) tools [28, 29] to identify copy number alterations (CNAs) in PANC tissues, but found no alteration in the miR-29c genomic region (Figure S2A). Furthermore, we assessed the methylation status of miR-29c in normal pancreatic tissues and PANC tissues by analyzing the publicly available data from TCGA (Figure S2B-a), finding that the methylation level detected by probe cy08855249 was higher in PANC tissues than in normal pancreatic tissues. Although the methylation level detected was inversely correlated with miR-29c expression levels (Figure S2B-b), it was not associated with PANC progression, which contradicted the earlier results (Figure 1B). Therefore, we suggest another mechanism reduces miR-29c in PANC. Additionally, GSEA showed remarkable correlation between miR-29c expression levels and the TGF-β-activated gene signatures (Figure 6A). Interestingly, TGF-β/Smad3 regulated miR-29 expression negatively [30]. The chromatin immunoprecipitation (ChIP) assay showed that endogenous Smad3 proteins bound to a sterol regulatory element (SRE) in the *MIR29C* promoter (Figure 6B); Figure 6C shows that miR-29c expression was decreased in PANC cells treated with TGF-β, but was increased in cells treated with a type I TGF-β receptor inhibitor or a neutralizing anti-TGF-β antibody. Furthermore, the luciferase activity of the Wnt signaling reporter was significantly increased in TGF-β-treated PANC cells, but was decreased in cells treated with a type I TGF-β receptor inhibitor or a neutralizing anti-TGF-β antibody (Figure 6D). Collectively, our data confirm that the TGF-β/Smad3 pathway decreases miR-29c expression by directly targeting the *MIR29C* promoter in PANC cells.

### MiR-29c expression correlated with Wnt cascade hyperactivation and Smad3 activity in clinical PANC

We examined whether activation of the TGF-β/Smad3/miR-29c/Wnt axis identified in our PANC cell models was also evident in clinical PANC. The miR-29c levels in 10 freshly collected PANC samples were inversely correlated with the mRNA levels of the following Wnt cascade downstream targets: *MYC* (*r* = −0.782, *P* = 0.008), *CD44* (*r* = −0.810, *P* = 0.004) and matrix metalloproteinase-7 (*MMP-7*, *r* = −0.888, *P* = 0.001); and four *bona fide* targets of miR-29c: *FRAT2* (*r* = −0.641 *P* = 0.046), *LRP6* (*r* = −0.667, *P* = 0.035), *FZD4* (*r* = −0.639, *P* = 0.047) and *FZD5* (*r* = −0.734, *P* = 0.016); and p-Smad3 (*r* = −0.812, *P* = 0.004) (Figure 6E). These data further support the notion that a hyperactive TGF-β/Smad3 pathway suppresses miR-29c expression, resulting in Wnt signaling activation and the consequent promotion of malignant PANC phenotypes, high tumor recurrence rate and poor prognosis of clinical PANC.

## DISCUSSION

An increasing amount of evidence shows that cancer treatments that fail to eradicate cancer stem cells (CSC) may lead to tumor recurrence, and these CSCs are implicated in tumor progression and metastasis, the critical causes of death in patients with cancer. Analysis of two public data sets revealed that miR-29c was decreased in patients who had died from PANC and in patients with proven tumor recurrence. We found that restoring miR-29c suppressed Wnt signaling in PANC cells, attenuated cell migration and invasion and stem cell-like phenotypes *in vitro* and *in vivo*. Next, we demonstrated that *FRAT2*, *LRP6*, *FZD4* and *FZD5* are *bona fide* targets of miR-29c. Furthermore, we determined that TGF-β/Smad3 signaling decreased miR-29c expression levels in clinical PANC samples. To summarize our findings, our study reveals that miR-29c regulates PANC migration, invasion and stem cell-like phenotypes via a novel mechanism, i.e. by modulating multiple Wnt cascade regulators, intimating that miR-29c might function as a tumor-suppressive miRNA in human PANC.

Clearly, a better understanding of the critical pathways involved in migration, invasion and stem cell-like phenotypes is important for identifying new molecular targets to eradicate PANC. One particularly significant pathway shown to correlate with self-renewal and metastasis is the Wnt/β-catenin signaling pathway. Indeed, more than 65% of pancreatic tumors show activation of the canonical Wnt/β-catenin pathway [22]. It has also been reported that nuclear β-catenin and cyclin D1 are co-overexpressed in the invasion front of colorectal cancer [31, 32]. In a breast cancer study, Lin and colleagues demonstrated that β-catenin modulated cyclin D1 expression [33]; later, Yook and associates found that the Wnt cascade regulates Snail1 activity [34]. The Wnt cascade can be divided into the canonical and non-canonical pathways. It is noteworthy that canonical Wnt signaling has been proven to regulate stemness, proliferation and differentiation in several adult stem cell niches [35-37]. Canonical Wnt signaling targets the receptor complex consisting of FZD family receptors and the LRP5/6 co-receptor, inducing the assembly of a FZD-Disheveled (DVL) complex and LRP5/6-AXIN-FRAT complex, leading to the stabilization and nuclear accumulation of β-catenin. Then, nuclear β-catenin engages the N-terminus of DNA-binding proteins of the TCF/lymphoid enhancing factor (LEF) family and modulates the transcription of a broad range of target genes. Although they have not been fully elucidated, several mechanisms activate the Wnt cascade, such as gene mutation, which interferes with β-catenin phosphorylation and ubiquitination [38-41], and the mutation-independent changes in the upstream effectors of β-catenin [42, 43]. Herein, we found that miR-29c directly targeted and suppressed the expression of four β-catenin upstream effectors (*FRAT2*, *LRP6*, *FZD4*, *FZD5*), thereby suppressing Wnt signaling and leading to decreased PANC cell migration, invasion and stem cell-like phenotypes.

An abundant class of small non-protein-coding RNAs that function as negative gene regulators, miRNAs play important roles in multiple biological processes, such as cellular differentiation, proliferation, angiogenesis, invasion, metastasis and oncogenesis [44]. Mounting evidence has shown that miRNA dysregulation correlates with virtually all human cancers and indicates that miRNAs can function as either tumor suppressors or oncogenes. The miR-29 family members (miR-29a, miR-29b, miR-29c), which differ at their last few 3′ end nucleotides, act as tumor suppressors and are downregulated in human cancers, such as aggressive chronic lymphocytic leukemia [45], colon cancer [46], non-small cell lung cancer [47] and nasopharyngeal carcinoma [48]. Interestingly, whether members of the same miRNA family control similar biological events remains unclear [49]. Nagano and colleagues reported that miR-29a functions as an oncomiRNA by activating the Wnt cascade [50]. However, we believe that downregulated miR-29c plays important roles in PANC cells. Reducing miR-29c not only promoted PANC cell stemness, but also PANC cell migration and invasion abilities by suppressing Wnt cascade inhibition both *in vitro* and *in vivo*. Significantly, restoring miR-29c greatly abrogated PANC cell aggressiveness. Our findings provide compelling support indicating that members of the same miRNA family can control different biological events.

Lastly, we showed that TGF-β/Smad3 regulated miR-29c expression negatively by binding to the *MIR29C* promoter, leading to the loss of miR-29c in PANC cells. Analysis of previously published gene expression profiles from PANC tissues indicated that the miR-29c reduction was not correlated with CNA or with promoter methylation level. TGF-β is a significant growth factor in PANC that promotes tumor growth and progression [51], and TGF-β/Smad3 signaling promotes renal fibrosis by inhibiting miR-29 [30]. Moreover, the GSEA showed that the miR-29c expression levels were correlated with the TGF-β-activated gene signatures in PANC cells. In this context, we found that Smad3 binding to the *MIR29C* promoter led to reduced miR-29c expression in PANC cells.

In summary, great effort has been expended to clarify the mechanisms underlying the aggressive nature of PANC, and decades of research have focused on screening the effectors involved in tumor-related biological processes. In the present study, we identified miR-29c as a major mediator of two hallmarks of aggressive PANC: limitless replicative potential, and tissue invasion and metastasis. These effects were significantly associated with the repression of four β-catenin upstream effectors, i.e. *FRAT2*, *LRP6*, *FZD4* and *FZD5*, resulting in constitutive activation of the Wnt cascade in PANC. Taken together, our study may provide new insights for understanding PANC cell migration and invasion as well as the stemness phenotype and therefore might contribute to future development of therapeutic interventions for this uniformly fatal disease.

## METHODS

### Cell lines

Normal *pancrease* cell line hTERT-HPNE and pancreatic cancer cell lines BxPC-3, Capan-1, Capan-2, HPAC, MIA PaCa-2, Panc 02.03, Panc 02.13 and PANC-1 were from ATCC (Manassas, VA, USA). The cells were grown in Dulbecco's modified Eagle's medium supplemented with 10% fetal bovine serum.

### Plasmids, virus production, and target cell infection

The human *MIR29C* gene was PCR-amplified from genomic DNA and cloned into a pMSCV-puro retroviral vector. MiR-29c sponge was constructed by annealing, purifying, and cloning oligonucleotides containing six tandem “bulged” miR-29c-binding motifs into the pMSCV vector. Human β*-catenin* were PCR-amplified from hTERT-HPNE complementary DNA (cDNA) and cloned into the pMSCV vector. The 3′UTRs of the human *FRAT2* (FRAT2-3′UTR), *LRP6* (LRP6-3′UTR), FZD4 (FZD4-3′UTR) and FZD5 (FZD5-3′UTR) genes, generated by PCR amplification from hTERT-HPNE, were cloned into the *Sac*I/*Xma*I sites of pGL3 luciferase reporter plasmid (Promega, Madison, WI, USA). Plasmid transfection was performed using Lipofectamine 2000 (Invitrogen, Carlsbad, CA, USA) according to the manufacturer's instructions. Stable cell lines expressing miR-29c and miR-29c sponge were generated via retroviral infection using HEK293T cells as described by Li *et al* [52] and selected with 0.5 μg/mL puromycin for 10 days.

### Western blotting analysis

Cells and tissues were harvested in sampling buffer [62.5 mmol/L Tris-HCl (pH 6.8), 10% glycerol, 2% sodium dodecyl sulfate (SDS)] and heated for 5 minutes at 100°C. Protein concentration was determined with the Bradford assay using a commercial kit purchased from Bio-Rad Laboratories (Hercules, CA, USA). Equal quantities of protein were separated electrophoretically on 10% SDS/polyacrylamide gels and transferred onto polyvinylidene difluoride membranes (Roche, Basel, Switzerland). The membranes were probed with diluted antibody. The expression of target proteins was determined with horseradish peroxidase-conjugated anti-rabbit immunoglobulin G (IgG)/anti-mouse IgG (Sigma-Aldrich, St Louis, MO, USA) and enhanced chemiluminescence (Pierce, Rockford, IL, USA) according to the manufacturers' suggested protocols. The membranes were stripped and reprobed with an anti-β-actin mouse monoclonal antibody (Sigma-Aldrich) as a loading control. The related antibodies were anti-phosphorylated(p)-Smad3, anti-E-cadhernin, anti-Vimentin, anti-β-catenin (Cell Signaling Technology, Beverly, MA, USA), anti-CD24, anti-CD44, anti-CD166, anti-EpCam, anti-EF-1, anti-p84 anti-FRAT2, anti-LRP6, anti-FZD4, anti-FZD5 (Abcam, Cambridge, MA, USA) and anti-CD133 (Epitomics, USA).

### RNA extraction and real-time quantitative PCR

Total miRNA from cultured cells and fresh surgical pancreatic cancer tissues was extracted using a mirVana miRNA Isolation Kit (Ambion, Foster City, CA, USA) according to the manufacturer's instructions. We synthesized cDNA using a TaqMan MicroRNA Reverse Transcription Kit (Applied Biosystems, Foster City, CA, USA) and quantified miR-29c expression using a miRNA-specific TaqMan MiRNA Assay Kit (Applied Biosystems). Real-time PCR was performed using the Applied Biosystems 7500 Sequence Detection System. MiRNA expression was defined based on the comparative threshold (Ct); relative expression levels were calculated as 2^(Ct miR-29c - Ct U6) after normalization with reference to the quantification of U6 small nuclear RNA expression.

### Microribonucleoprotein immunoprecipitation assay

Cells were cotransfected with a plasmid that encodes hemagglutinin-tagged (HA)-Ago1 and miR-29c (100 nM), followed by HA-Ago1 immunoprecipitation (IP) using an anti-HA antibody. Real-time PCR analysis of the IP material was used to test the association of *FRAT2*, *LRP6*, *FZD4*, *FZD5, DNMT3A* and glyceraldehyde-3-phosphate dehydrogenase (*GAPDH*) mRNA with the RNA-induced silencing complex.

### Luciferase assay

Cells (3 × 10^3^) were seeded in triplicate in 48-well plates and allowed to settle for 24 hours. Luciferase reporter plasmids (100 ng) or 100 ng control luciferase plasmid plus 1 ng pRL-TK *Renilla* plasmid (Promega) were transfected into pancreatic cancer cells using Lipofectamine 2000 (Invitrogen). Luciferase and *Renilla* signals were determined 24 hours after transfection using a Dual Luciferase Reporter Assay Kit (Promega).

### Cell invasion assay

Glioblastoma cells (2 × 10^4^) were plated on the top side of a polycarbonate Transwell filter (with Matrigel) in the top chamber of a BioCoat Invasion Chamber (BD Biosciences, Bedford, MA, USA) and incubated at 37°C for 22 hours; cells in the top chamber were removed with cotton swabs. Cells that had migrated and invaded to the lower membrane surface were fixed in 1% paraformaldehyde, stained with hematoxylin, and counted under a microscope (10 random fields per well, ×100 magnification). The cell counts were expressed as the mean number of cells per field.

### Flow cytometric analysis

Cells were dissociated with trypsin and resuspended at 1 × 10^6^ cells per milliliter in DMEM containing 2% FBS and then preincubated at 37°C for 30 minutes with or without 100 μM verapamil (Sigma-Aldrich) to inhibit ABC transporters. The cells were subsequently incubated for 90 minutes at 37°C with 5 μg/ml Hoechst 33342 (Sigma-Aldrich). Finally, the cells were incubated on ice for 10 minutes and washed with ice-cold PBS before flow cytometric analysis. The data were analyzed by Summit 5.2 software (Beckman Coulter).

### Tumor xenografts

The nude mice were randomly divided into 4 groups (n = 12 per group). Indicated cells of 4 dosages (1 × 10^6^, 1 × 10^5^, 1 × 10^4^, 1 × 10^3^) were inoculated with Matrigel (final concentration of 25%) into the inguinal folds of nude mice. The mice were scarified 31 days after inoculation and the tumors were excised and subjected to pathologic examination.

### Microarray data processing and visualization

Microarray data were downloaded from The Cancer Genome Atlas (TCGA) database (http://cancergenome.nih.gov/). Analysis of miR-29c expression in PANC tissues compared with that in normal pancreas tissues was using a published microarray-based high-throughput assessment (n=148, *P*<0.001; NCBI/GEO/GSE24279). The data were downloaded from NCBI and analyzed using SPSS16.0 software. Gene Set Enrichment Analysis (GSEA) was performed using GSEA 2.0.9 (http://www.broadinstitute.org/gsea/).

### Statistics

All statistical analyses were carried out using the SPSS 16.0 statistical software package (SPSS Inc., Chicago, IL, USA). The χ^2^ test was used to analyze the relationship between QKI expression and clinicopathological characteristics. Bivariate correlations between study variables were calculated by Spearman's rank correlation coefficients. *P* < 0.05 was considered statistically significant.

## SUPPLEMENTARY MATERIAL FIGURES


