# Supplementary material for: Reduction of miR-29c enhances pancreatic cancer cell migration and stem cell-like phenotype
Source: Oncotarget. 2014 Dec 30;6(5):2767–78. doi: 10.18632/oncotarget.3089 (PMC4413616; doi:10.18632/oncotarget.3089)
Supplement: Supplementary file 1 [file oncotarget-06-2767-s001.pdf]

Reduction of miR-29c enhances pancreatic cancer cell migration and stem cell–like phenotype

Supplementary Material

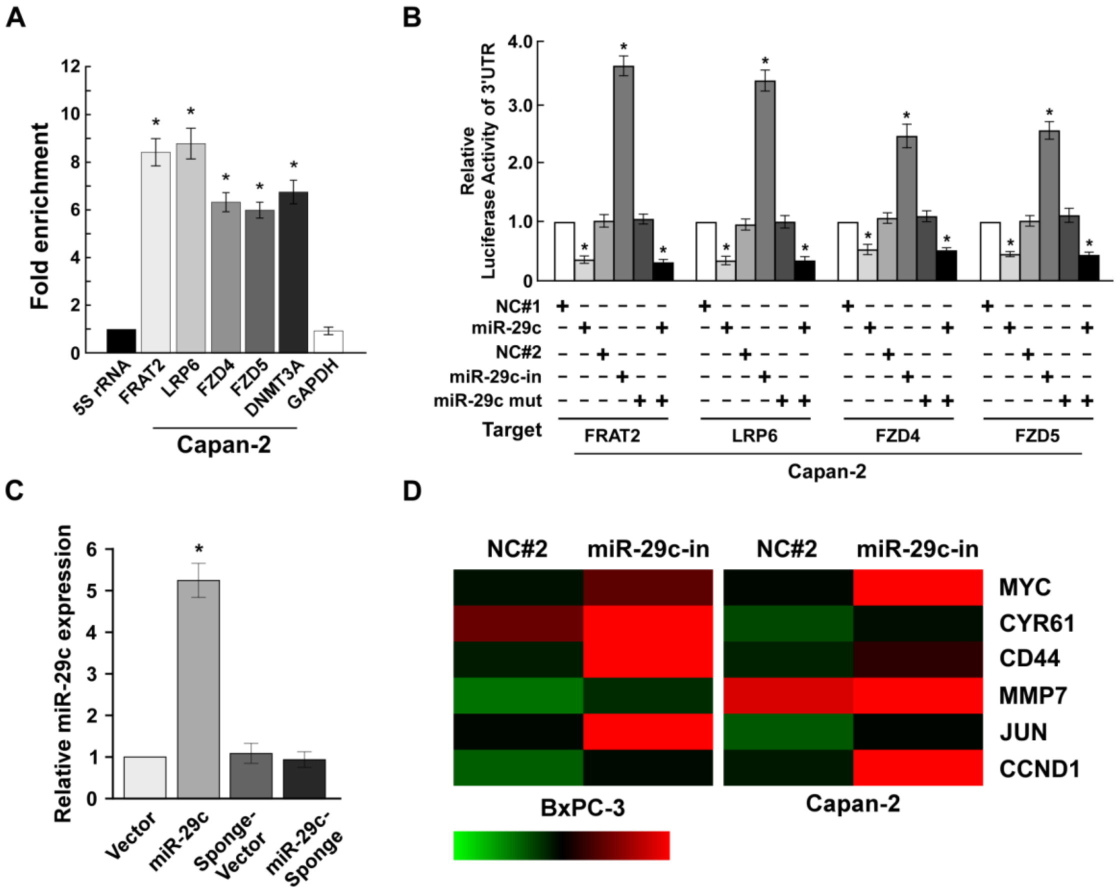

Supplemental Figure 1: A, miRNP IP assay showed association of miR-29c with *FRAT2*, *LRP6*, *FZD4*, *FZD5*, *DNMT3A* and *GAPDH* were used as positive and negative controls, respectively, and *5S* rRNA was used as a control for overall expression levels. **B**, Luciferase activities of FRAT2-3′UTR, LRP6-3′UTR, FZD4-3′UTR or FZD5-3′UTR in vector- or miR-29c-transduced cells, or in miR-29c-transduced cells transfected with miR-29c-mut, or in vector-transduced cells transfected with NC or miR-29c inhibitor. **C,** Real-time PCR analysis of miR-29c expression in the indicated samples of nude mouse tissues. D, Relative mRNA expression of Wnt/β-catenin-regulated genes in the indicated cells was assessed by real-time PCR. *GAPDH* was used as a loading control.

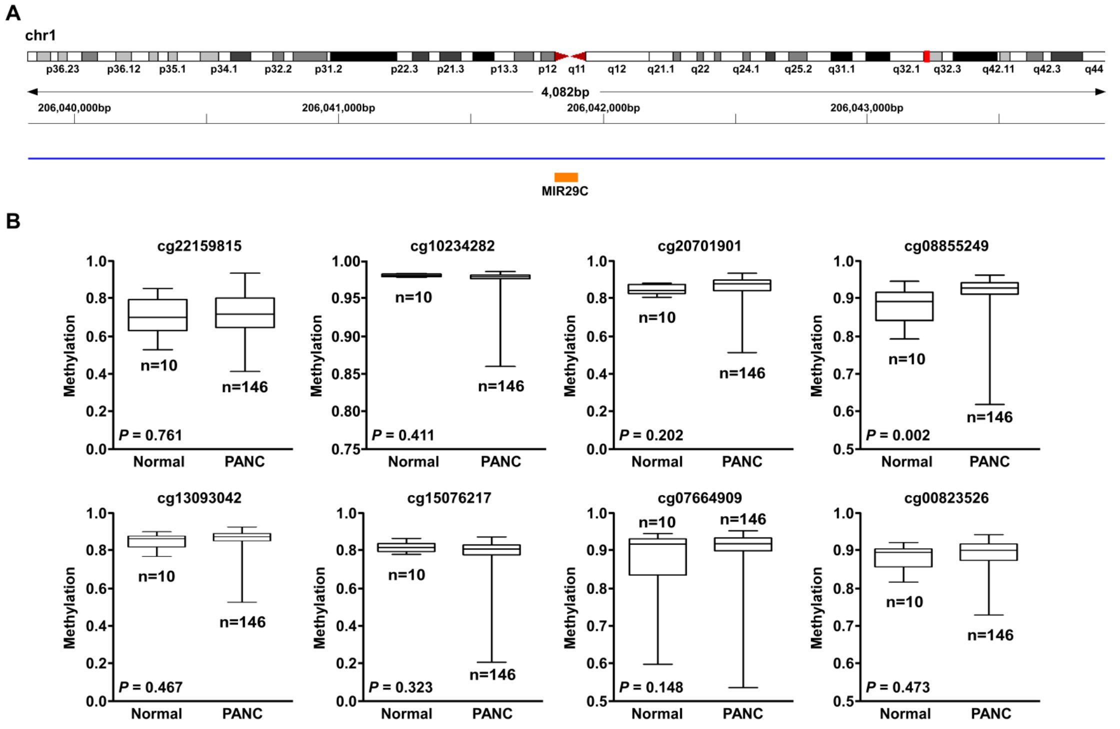

		cg22159815	cg10234282	cg20701901	cg08855249	cg13093042	cg15076217	cg07664909	cg00823526
MIR29C	Pearson Correlation	-0.066	-0.026	-0.092	-0.366	-0.196	-0.075	-0.020	-0.087
	Sig. (2-tailed)	0.523	0.804	0.374	0.000	0.056	0.466	0.845	0.398
	N	96	96	96	96	96	96	96	96

Supplemental Figure 2: A, GISTIC analysis of copy number aberration of 185 pancreatic cancer cases from the TCGA database; the straight blue bar showed no changes in the genomic region within miR-29c. **B**, Methylation status of miR-29c between normal pancreas tissues and PANC tissues via analysis of the publicly available data from the TCGA database. Statistical analysis showed a correlation between miR-29c expression and methylation status.

Clinicopathological characteristics of 132 pancreatic cancer patients

	No.	(%)
**Age (years)**		
≤45	7	(5.3)
＞45	125	(94.7)
**Clinical stage**		
I	16	(12.1)
II	96	(72.7)
III	13	(9.9)
IV	7	(5.3)
**T classification**		
**x**	2	(1.5)
1	18	(13.6)
2	32	(24.3)
3	80	(60.6)
**N classification**		
x	14	(10.6)
0	101	(76.5)
1	17	(12.9)
**Distant metastasis**		
Yes	7	(5.3)
No	125	(94.7)
**Vital status (at follow-up)**		
Alive	9	(6.8)
Death because of pancreatic cancer	121	(91.7)
Death because of other than pancreatic cancer	2	(1.5)
